# The impact of the HbA1c level of type 2 diabetics on the structure of haemoglobin

**DOI:** 10.1038/srep33352

**Published:** 2016-09-14

**Authors:** Shaoying Ye, Ping Ruan, Junguang Yong, Hongtao Shen, Zhihong Liao, Xiaolei Dong

**Affiliations:** 1 Department of Occupational and Environmental Health, Guangdong Pharmaceutical University, Guangzhou, China; 2 Department of Biomedical Engineering, Guangdong Pharmaceutical University, Guangzhou, China; 3 Department of Endocrinology, the affiliated outpatient department, Guangdong Pharmaceutical University, Guangzhou, China; 4 College of Physics and Technology, Guangxi Normal University, Guilin, China; 5 Department of Endocrinology, the First Affiliated Hospital, Sun Yat-sen University, Guangzhou, China

## Abstract

This study explores the impact of HbA_1_c levels on the structure of haemoglobin (Hb) in patients with type 2 diabetes. Seventy-four diabetic patients were classified into the following two groups based on their level of HbA_1_c: group A, patients with good glycaemic control (HbA_1_c < 7.0%, n = 36); group B, patients with persistent hyperglycaemia (HbA_1_c ≥ 9.0%, n = 38). Thirty-four healthy people served as controls (group H). Hb structure was examined by Fourier transform infrared spectroscopy (FTIR), and diabetic erythrocytes were modelled to estimate the impact of glucose on these cells and Hb. Increasing glucose concentrations altered both erythrocyte parameters and the Hb secondary structure. Group B differed significantly from group H (p < 0.05): in the former, the ordered Hb secondary structure had a strong tendency to transform into a disordered secondary structure, decreasing structural stability. We presumed here that high HbA_1_c levels might be a factor contributing to Hb structural modifications in diabetic patients. FTIR spectral analysis can provide a novel way to investigate the pathogenesis of type 2 diabetes mellitus.

Although the pathogenesis of type 2 diabetes mellitus is unclear, the increasing prevalence of this disorder is of great concern and constitutes a global public health crisis. HbA_1_c is the gold standard for evaluating the long-term glycaemic control of diabetic patients because it accurately reflects real glycaemic levels *in vivo*^1^,^2^. The American Diabetes Association (ADA) treatment guidelines suggest an HbA_1_c level <7.0% as the primary glycaemic control target for diabetics^3^, and a decrease in HbA_1_c level reduces the prevalence of chronic complications due to the disease^4^. Monnier *et al*.^5^ found the concentration of products of oxidative stress to correlate positively with an increasing HbA_1_c level. Furthermore, haemodynamic changes in diabetic patients, which can cause both macrovascular and microvascular disease^6^,^7^, correlate strongly with increasing HbA_1_c levels and play an important role in the pathogenesis of diabetes^8^.

Haemoglobin (Hb) is the most important erythrocyte protein, and structural variations in Hb affect the function of these cells as well as vasoconstriction capacity. Environmental changes, temperature increases, and pH deviations, together with chemical modifications, can induce structural and functional alterations in Hb^9^,^10^,^11^. Moreover, a disordered Hb conformation or Hb aggregation in erythrocytes, resulting from oxidative stress in diabetic patients, accelerates the development and progression of diabetes and its complications^12^,^13^,^14^. As a potential tool for diabetes mellitus diagnosis, Scott *et al*.^15^ applied Fourier transform infrared spectroscopy (FTIR) to characterize molecular signatures in saliva from diabetic patients. FTIR has also been used to identify alterations in the spectral parameters of serum and tissues from diabetic patients^16^,^17^. To date, however, few quality-related studies have addressed the impact of the HbA_1_c level of diabetic patients on the structure of Hb. Therefore, in this study, we utilized FTIR to evaluate the influence of elevated HbA_1_c levels on the structure of Hb in type 2 diabetic patients. In addition, a diabetic erythrocyte model was used to estimate the impact of glucose levels on erythrocytes and Hb.

## Materials and Methods

### Subjects

Seventy-four adults with type 2 diabetes mellitus from the Endocrinology Department of the First Affiliate Hospital of Guangdong Pharmaceutical University, China, were enrolled, as were 34 non-diabetic, age-matched, healthy adult controls [n = 34, 17 females/17 males, mean age 52.18 ± 9.23 years] from the Out-patient Department of the First Affiliate Hospital of Guangdong Pharmaceutical University, China (group H). The study was approved by the Ethics Committee of the First Affiliate Hospital of Guangdong Pharmaceutical University, China, and was conducted in accordance with the ethical guidelines of the 1975 Declaration of Helsinki. Written informed consent was obtained from all participants. Patients with type 2 diabetes mellitus were classified into two groups based on their HbA_1_c levels. Patients with an HbA_1_c level <7.0% were enrolled as group A, a group with good glycaemic control [n = 36, 18 females/18 males, mean age 53.75 ± 14.54 years]; group B comprised patients with sustained hyperglycaemia and an HbA_1_c level ≥9.0% [n = 38, 18 females/20 males, mean age 55.17 ± 12.45 years]. The inclusion criteria for all subjects were as follows: (1) patients who were in accordance with the diagnostic criteria for diabetes (WHO-1999); (2) patients without acute or chronic diabetic complications; (3) patients without cancer or serious infectious diseases; (4) patients without renal, cardio- or cerebro-vascular complications or digestive system diseases; (5) patients without haematological disorders; and (6) patients who had not used antibiotics or immunosuppressive or antiplatelet aggregation drugs in the preceding month.

### Collection of blood samples

Blood was collected by venepuncture after a 12 h overnight fast into a vacutainer with EDTA-K2. The HbA_1_c level was measured by high-performance liquid chromatography, and the fasting blood glucose level was determined using a glucose oxidase method.

### *In vitro* preparation of a hyperglycemic erythrocyte model

To model the impact of glucose on erythrocyte parameters and Hb, erythrocytes from healthy adults were prepared as described by Dong^18^. Briefly, intact erythrocytes were incubated with different concentrations of glucose (8.5, 15, and 30 mM) in 50 mM phosphate-buffered saline (PBS, pH 7.4) in capped vials under sterile conditions at 37 °C for 7 days. Control erythrocytes were similarly prepared and incubated without glucose. After incubation, the samples were dialyzed extensively against 50 mM phosphate buffer (pH 7.4) to remove the excess glucose. The samples were stored at 4 °C until use.

### Preparation of human Hb

In total, 1.5 ml of anticoagulated whole blood was centrifuged at 2000 rpm for 10 min, and the clear plasma was discarded. The packed erythrocytes were washed with 0.9% NaCl (10 ml per ml of packed RBC) and centrifuged at 3500 rpm for 5 min; the supernatant was discarded. This wash cycle was performed three times. After the last wash, 10 ml of precooled distilled water was added per ml of packed RBC, and the suspension was vortexed for 1 min. The mixture was stored at 4 °C overnight to release Hb completely and then centrifuged at 12,000 rpm for 45 min at 4 °C. The supernatant was first filtered through a 0.22 μm sterile membrane and then purified by gel filtration chromatography with a Sephadex G-75 resin column. The Hb fraction was measured by SDS-PAGE to ensure >95% purity. After several hours of dialysis against distilled water for desalination, the purified Hb was freeze-dried and stored at −20 °C until use. Hb from the modelled diabetic erythrocytes was prepared in a similar manner.

### Detection of erythrocyte parameters and Hb

Morphology alterations of erythrocytes were detected by the new microscopic static imaging and analysis technique. Hb was prepared as described above and measured by Fourier Transform Infrared Measurement (FTIR).

### Fourier transform infrared spectroscopy (FTIR)

To prepare 1 mm KBr pellets for analysis, approximately 1.0~1.5 mg of lyophilized Hb was mixed with dry KBr at a mass ratio of 1:100. Infrared spectra were obtained using an infrared spectrometer (Bruker, Tensor 37, German) equipped with an air-cooled DTGS detector and an effective resolution of 4 cm^−1^. Each spectrum was acquired for 64 sample interferograms recording in the ~4000–400 cm^−1^ region at room temperature (25 °C). The interfering spectra of air and water vapour were recorded as background and subtracted automatically by the software. Three spectra from each sample were obtained and averaged using OPUS 7.0 software to obtain a final average group spectrum normalized to specific bands for visual demonstration in the figures. An eleven-point Savitsky-Golay smooth function was used to remove noise from the spectrum. The second-derivative spectra were obtained using Peak Fit V4.0 software to ascertain the number and positions of peaks in the Amide I region of Hb for Fourier self-deconvolution and curve-fitting analysis. After baseline correction, the best fit for decomposing the Amide I band in the region between 1700 cm^−1^ and 1600 cm^−1^ was obtained by Gaussian curve-fitting. The fit was converged with a correlation (R^2^) of ~0.999–1 and a standard error of 0.005.

### Statistical analysis

Normally distributed data are displayed as the mean ± SD. The statistical significance of between–group comparisons was determined by ANOVA. Abnormally distributed data are shown as the median; these data were analysed using the rank-sum test. A value of p < 0.05 was considered statistically significant.

## Results

### General clinical characteristics

There were no significant differences in age or gender between the controls (group H) and patients with diabetes (groups A and B). The BMI, fasting blood glucose (FPG) and HbA_1_c level of groups A and B were significantly higher (p < 0.05) than those of the controls. Between the diabetic groups, the FPG and HbA_1_c levels of group B were significantly higher (p < 0.05) than those of group A, but there was no significant difference in BMI. These data are shown in Table 1.

### Analysis of the hyperglycemic erythrocytes model

**Figure 1:**
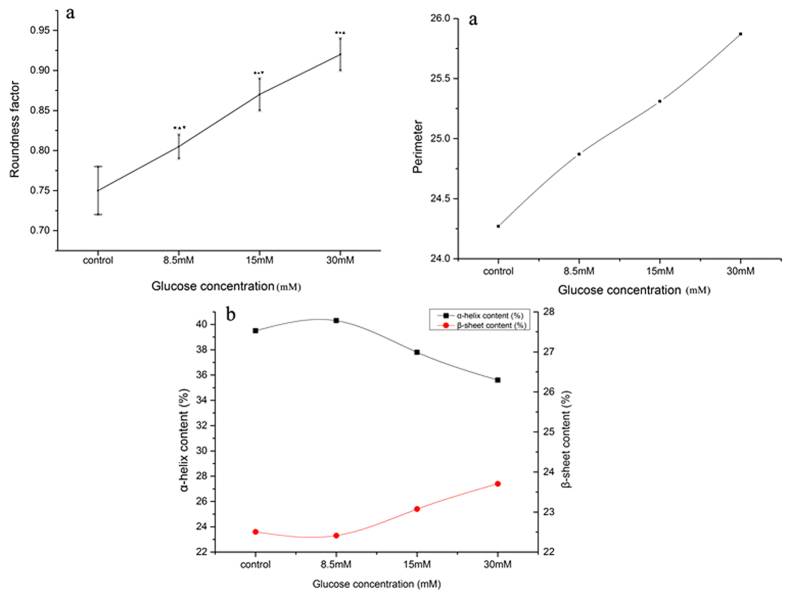
Variation of erythrocyte parameters (**a**) and secondary structures of Hb (**b**) in glucose buffers at 8.5, 15, and 30 mM compared with PBS.

As the glucose concentration increased, the parameters of the modelled hyperglycemic erythrocytes were significantly enhanced. The effect of glucose on the secondary structure of Hb was analysed by FTIR, and Fig. 1 shows the changes in secondary structure of Hb incubated in buffers containing different glucose concentrations compared with PBS. The Hb secondary structure showed a decreased α-helix content at 15 mM and 30 mM glucose, whereas it increased slightly at 8.5 mM. The β-sheet content showed a tendency to increase in parallel with the glucose concentration.

### Analysis of erythrocyte morphology in diabetic patients

**Figure 2:**
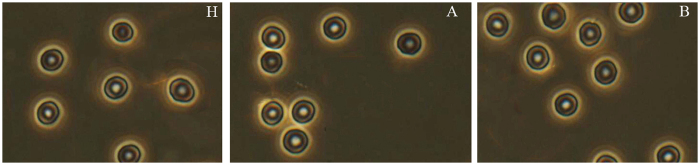
Morphology alterations of erythrocytes in normal (**H**) and diabetic groups (**A,B**).

As shown in Fig. 2, the long axis of erythrocytes in groups A and B have gotten shorten along with the increasing HbA_1_c level, which have made the morphology of erythrocyte from oval gradually became into near-circular. It implied that the rigidity of erythrocyte have significantly increased and result in a decrease in the erythrocyte deformability.

### FTIR spectra of Hb

**Figure 3:**
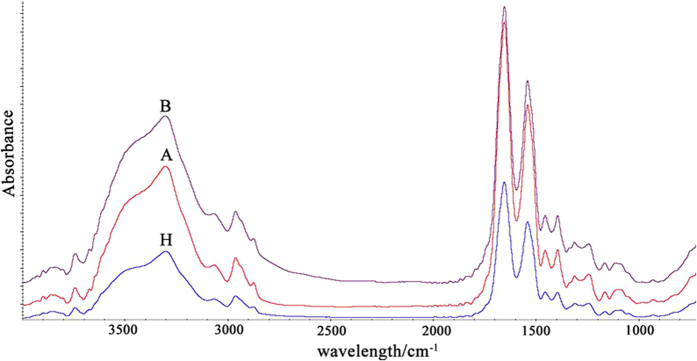
Typical FTIR spectra of normal (**H**) and diabetic patients’ Hb (**A,B**).

The FTIR spectra of Hb in the ~4000–400 cm^−1^ region for both the control (group H) and diabetic patients (groups A and B) are shown in Fig. 3. The detailed spectral band assignments of the observed peaks in all of the samples are labelled and presented in Table 2. As shown in Fig. 3 and Table 2, the spectrum of an Hb sample is composed of various peaks originating from the contributions of different functional groups of the protein. However, as presented in Fig. 3, there were no obvious differences between the position and number of characteristic bands for the control and diabetic groups. The observed differences in intensity among the three groups might result largely from the different concentration of the samples in the KBr pellets. The FTIR spectra of Hb exhibited a number of amide bands, with Amide I, II and III bands at 1654 cm^−1^, 1541 cm^−1^ and 1312 cm^−1^ respectively. These bands can represent a superimposition of conformational structures and changes in Hb. The Amide I band (1700–1600 cm^−1^) was selected for further analysis because it is the most sensitive to secondary structural changes in Hb.

### Comparison of relative intensity ratios among the three groups

The relative intensity ratios of the different peaks can reflect variations in the quantity of different functional groups in samples. It can also be used to eliminate the influence of uneven thicknesses and weighing errors of sample pellets. Because the peaks of FTIR spectra in the region above 3300 cm^−1^ and below 1000 cm^−1^ are seriously affected by the absorption of H_2_O and CO_2_, respectively, the infrared absorption data of these two regions were not analysed. Accordingly, the peaks of characteristic bands in the ~3303 cm^−1^–1000 cm^−1^ region were used to calculate the relative intensity ratios with adjacent peaks; the results are summarized in Table 3. There were no significant differences in the relative intensity ratios of I_3303_/I_3306_, I_1456_/I_1396_, I_1313_/I_1254_, or I_1166_/I_1089_ (I = peak intensity) among the three groups. Although the relative intensity ratios of Hb at I_2960_/I_2874_ and I_1654_/I_1541_ were slightly lower in group A than in group H, this did not reach statistical significance (p > 0.05). In group B however, these two relative intensity ratios were reduced to 2.08 and 1.28, which were significantly lower than those of groups A or H (p < 0.05). We inferred from these findings that the structure of Hb in group B may have changed.

### Qualitative analysis of the Amide I band

**Figure 4:**
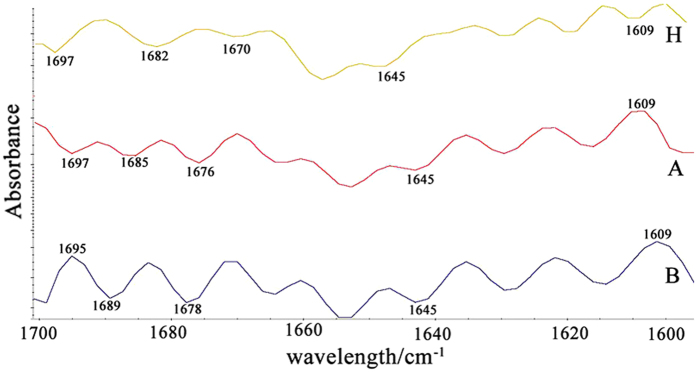
Representative secondary derivative mean FTIR spectra of normal Hb (H) and diabetic groups (A and B), in the Amide I band.

The Amide I band is a broad peak formed by the superimposition of the underlying secondary structure of proteins, such as helices, beta sheets, and other conformations, in the original FTIR spectra. The exact position of the underlying secondary structure and information regarding some specific inconspicuous structures, can be visualized and highlighted by their second-derivative spectra. Second-derivative spectra were therefore used to reveal the underlying components in the Amide I bands for the three subject groups. As shown in Fig. 4, there were obvious differences in the number, position, signal intensity and pattern of the underlying components among the three groups. Compared with group H, the signal intensity and pattern of peaks located at 1609 cm^−1^, 1645 cm^−1^, 1670 cm^−1^, 1682 cm^−1^ and 1697 cm^−1^ differed significantly between groups A and B. The negative peak at 1609 cm^−1^ in group H was transformed into a positive peak in groups A and B. In addition, the peaks at 1670 cm^−1^ and 1682 cm^−1^ of groups A and B were both blue shifted and had a significantly higher intensity compared to group H. The pattern of the peak at 1697 cm^−1^ in group B changed and was red shifted to 1695 cm^−1^ with a higher intensity compared to group H. With increasing HbA_1_c level, there was a significant increase in the intensity of the peak located at 1645 cm^−1^ among the three groups (p < 0.05).

### Quantitative analysis of the Amide I band

The underlying secondary structures of Hb in the Amide I band were revealed by Gaussian curve-fitting analysis combined with Fourier self-deconvolution and the second derivative. The proportion of each secondary structure average area corresponds to the content of the different secondary structural components of Hb. The results of the curve-fitting analysis based on the Amide I band are summarized in Table 4 (α-helix, β-sheet, β-turns, and random coil). All the secondary structures for the controls and diabetic patients were subjected to pairwise comparisons. When compared to group H, group A showed slight changes in the content of α-helix and β-sheet structures that did not reach statistical significance (p > 0.05). Group B showed a significant reduction in the content of α-helix structures (p < 0.05), whereas the content of β-sheet structures was significantly increased (p < 0.05). Compared with group A, group B showed a significant increase in the content of β-sheet structures associated with a decrease in α-helix structures as the HbA_1_c level increased beyond 9.0% (p < 0.05).

## Discussion

Type 2 diabetes mellitus is a complex endocrine disease associated with an extensive list of complications that ultimately lead to a cluster of disorders. The concentration of HbA_1_c is significantly increased with ambient hyperglycaemia and as it reflects the extent and management of a patient’s diabetes, also predicts the risk for complications. The aggregation of unfolded proteins could result in the so-called amyloidosis or amyloid-like disease of type 2 diabetes mellitus^19^. Pathological changes in the cellular milieu could cause conformational changes including aggregation and stable or unstable polymerization that would render a protein non-functional. Such events may even cause intracellular toxicity^20^,^21^,^22^,^23^.

We found that the parameters of erythrocytes changed significantly as the glucose concentration increased, in agreement with Dong^18^, who studied red blood cell deformation in type 2 diabetics. Moreover, the secondary structure of Hb had a strong tendency to change with increasing glucose concentration. Thus, we propose that high glucose concentrations may affect erythrocytes and Hb. Our study evaluated the impact of the HbA_1_c level on the structure of Hb in type 2 diabetic patients. The results indicated that the relative intensity ratios of Hb in group A were slightly but not significantly lower than those of group H, arguing against structural alterations in Hb intramolecular bonding at an HbA_1_c level below 7.0%. A well-controlled blood glucose environment may have little impact on the structure of Hb. However, the relative intensity ratios of Hb at I_2960_/I_2874_ and I_1654_/I_1541_ in group B were significantly lower than those of group H or A. The significant decline in the relative intensity ratio, which reflects alterations in the content of different functional groups, may signify intramolecular bonding variations that affect structure in Hb. The peaks at 2961 cm^−1^ and 2874 cm^−1^ originate from asymmetric and symmetric CH_3_ stretching vibrations, respectively, of side chains in Hb. The peak at 1654 cm^−1^ is the Amide I band of Hb that mainly corresponds to C=O stretching vibrations of peptide linkages in Hb, and the peak located at 1541 cm^−1^ is the Amide II band that primarily derives from N-H and C-N stretching vibrations. Our results resemble those of Arif *et al*.^24^, who characterized human serum albumin in type 1 diabetic patients. It is highly probable that the normal circulation milieu would change in patients whose HbA_1_c level is greater than 9.0% for prolonged periods of time. Ambient hyperglycaemia *in vivo* may allow glucose molecules to circulate as free aldehydes and participate in Hb glycosylation, changing intramolecular bonding and affecting the conformation.

Secondary structural elements play an important role in the conformation of a protein. An α-helix structure is mainly formed by intramolecular hydrogen bonding, whereas β-sheet structures are formed by intermolecular hydrogen bonding. As protein insolubility increases in proportion to the content of β-sheet structures, proteins show a strong tendency to aggregate with significant increases in β-sheet content^25^. Previous studies have shown that environmental changes may shift the equilibrium of a protein’s conformation and distort its secondary structure^23^. However, our study found no significant differences in Hb secondary structural content between groups A and H, though the Hb secondary structural content of group B clearly differed from that of groups A and H. Statistical analysis suggested that changes in the secondary structure of Hb might be associated with an HbA_1_c level greater than 9.0%. Mahmoud *et al*.^26^ previously reported that as the glycaemic level increased to 285 mg/dl, the content of β-sheet structures in diabetic patients increased to reduce the solubility of erythrocyte membrane proteins and erythrocyte mobility.

Our findings suggest that significant increases in the content of β-sheet structures and reductions in α-helix structures occurred in group B as the HbA_1_c level rose above 9.0%. It is highly probable that Hb exposed to high glucose in diabetics with elevated HbA_1_c levels becomes chemically modified, transforming α-helices into β-sheet structures and increasing the content of unfolded forms of the protein. These secondary structural variations are presumed to lead to conformational changes in Hb^13^,^14^,^27^,^28^. Native Hb is a tightly folded tetrameric globular protein; however, increases β-sheet content unfolds the structure, affecting transition of the spatial conformation and decreasing structural stability. Previous studies report that glucose-induced glycation of Hb exposed to a persistent hyperglycaemic environment promotes conformational changes and also affects Hb’s affinity for oxygen^29^,^30^. *in vivo*, an elevated HbA_1_c level stimulates a glucose to fructose shunt via the polyol pathway and increases the concentration of fructose^31^, and Tania *et al*.^29^ demonstrated fructation-induced structural and functional modifications of Hb. Both glucose and fructose in blood may be presumed to interact with Hb to induce conformational changes, and the structural unfolding of Hb may induce haeme degradation or displacement^32^, which is highly likely to weaken the affinity of Hb for oxygen.

Hb is the major component of erythrocytes; thus, when the HbA_1_c level is elevated, this ambient hyperglycaemia may increase the β-sheet structure content of Hb, causing it to aggregate. The consequent decrease in solubility of Hb in erythrocytes could increase the viscosity of the contents of these cells. Such changes may reduce the deformability of the erythrocytes of diabetic patients^33^ and impair erythrocyte flexibility, which may adversely affect the microcirculation and lead to diabetic complications by impeding their flow through capillaries^34^,^35^,^36^. Furthermore, structural changes in Hb that might be induced by persistent hyperglycaemia may decrease the peroxidase activity of Hb^29^. Overall, the impaired structure and function of erythrocytes in diabetes might be associated with the development of vascular changes^37^,^38^.

The secondary structure of Hb in diabetic patients with good glycaemic control (HbA_1_c < 7.0%) was not significantly altered, though distinct alterations did appear at HbA_1_c elevations above 9.0%. We therefore presume that an elevated HbA_1_c level might be a factor contributing to Hb structural modifications in diabetics. Structural changes in Hb may have deleterious effects linked to the pathological complications of type 2 diabetes mellitus. However, our present study is limited and further researches are needed to demonstrate and support our findings.

## Additional Information

**How to cite this article**: Ye, S. *et al*. The impact of HbA1c level of type 2 diabetics on the structure of haemoglobin. *Sci. Rep.* **6**, 33352; doi: 10.1038/srep33352 (2016).

## Figures and Tables

**Table 1:** Baseline demographics of the control (H) and diabetic groups (A,B).

Characteristics	Group H	Group A	Group B
Number	34	36	38
Sex(females/males)	17/17	18/18	18/20
Age(years)	52.18 ± 9.23	53.75 ± 14.54	55.17 ± 12.45
BMI(kg/m2)	21.56 ± 1.05	24.18 ± 3.21*	24.67 ± 3.19*
FPG(mmol/l)	5.27 ± 0.621	7.21 ± 1.44*	10.21 ± 2.58*^△^
HbA1c(% mmol/mol)	4.69 ± 0.31	6.05 ± 0.46*	11.93 ± 1.10*^△^

Data are expressed as the mean ± SD. Group H: healthy controls; Group A: patients with good glycaemic control (HbA_1_c < 7.0%, 53 mmol/mol); Group B: patients with persistent hyperglycaemia (HbA_1_c ≥ 9.0%, 75 mmol/mol); BMI: body mass index; FPG: fasting plasma glucose.

*p < 0.05, compared with group H, ^△^p < 0.05, compared with group A.

**Table 2:** General band assignments of FTIR spectra of Hb.

Wavelength/cm^−1^	Definition of the spectral assignment
3303	Amide A (mainly N-H stretching)
3061	Amide B (N-H bending)
2961	CH_3_ asymmetric stretching
2874	CH_3_ symmetric stretching
1654	Amide I (80% C=O stretching, 10% N-H bending, 10% C-N stretching)
1541	Amide II (60% N-H bending, 40% C-N stretching)
1453	CH_2_ bending
1396	CH_3_ symmetric stretching
1312	Amide III
1243	Amide III
1165	CO-O-C asymmetric stretching
1088	C-O stretching
932	C-C-(N) stretching

**Table 3:** Comparison of FTIR spectrum absorption peaks among the groups 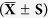 .

Relative intensity ratio	Group H	Group A	Group B
I_3303_/I_3061_	4.51 ± 0.416	4.49 ± 0.183	4.37 ± 0.383
I_2960_/I_2874_	2.26 ± 0.149	2.20 ± 0.108	2.08 ± ± 0.183*^△^
I_1654_/I_1541_	1.39 ± 0.185	1.36 ± 0.660	1.28 ± 0.859*^△^
I_1456_/I_1396_	1.04 ± 0.090	1.04 ± 0.072	1.07 ± 0.082
I_1313_/I_1254_	1.06 ± 0.255	1.09 ± 0.391	1.07 ± 0.518
I_1166_/I_1089_	0.833 ± 0.50	0.832 ± 0.34	0.830 ± 0.69

Group H: healthy controls; Group A: patients with good glycaemic control (HbA_1_c < 7.0%, 53 mmol/mol); Group B: patients with persistent hyperglycaemia (HbA_1_c ≥ 9.0%, 75 mmol/mol).

*p < 0.05, compared with group H, ^△^p < 0.05, compared with group A.

**Table 4:** Quantitative estimation of haemoglobin secondary structure from control and diabetic subjects 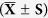.

Secondary structure	Percentage of content (%)		
	Group H(n = 34)	Group A(n = 36)	Group B(n = 38)
α-helix	38.52 ± 1.82	36.30 ± 2.56	29.48 ± 2.34*^△^
β-sheet	24.41 ± 2.98	26.98 ± 3.07	36.98 ± 2.21*^△^
β-turn	23.74 ± 3.41	23.50 ± 3.21	18.04 ± 3.13
Random coil	13.33 ± 4.67	13.22 ± 4.05	15.50 ± 4.22

Group H: healthy controls; Group A: patients with good glycaemic control (HbA_1_c < 7.0%, 53 mmol/mol); Group B: patients with persistent hyperglycaemia (HbA_1_c ≥ 9.0%, 75 mmol/mol).

*p < 0.05, compared with group H, ^△^p < 0.05, compared with group A.
